# Fitness Adaptations to a Combined Strength and Aerobic Training Program During the Transition Period in Young Soccer Players

**DOI:** 10.3390/sports14030088

**Published:** 2026-02-26

**Authors:** Yiannis Michailidis, Andreas Stafylidis, Athanasios Mandroukas, Konstantinos Georgiadis, Georgios Karamousalidis, Georgios Antoniou, Angelos E. Kyranoudis, Eleni Semaltianou, Vasilios Mittas, Thomas I. Metaxas

**Affiliations:** 1 Laboratory of Evaluation of Human Biological Performance, Department of Physical Education and Sports Sciences, Aristotle University of Thessaloniki, University Campus of Thermi, 57001 Thessaloniki, Greece; astafylidis@phed.auth.gr (A.S.); amandrou@phed.auth.gr (A.M.); akyran@phed.auth.gr (A.E.K.); esemalt@phed.auth.gr (E.S.); tommet@phed.auth.gr (T.I.M.); 2 Department of Physical Education and Sport Science, Aristotle University of Thessaloniki, 62500 Serres, Greece

**Keywords:** aerobic capacity, VO_2_max, body weight, body fat, jumping ability, countermovement jump, squat jump, football, youth

## Abstract

The annual soccer training cycle consists of preparatory, competitive, and transition periods. The transition phase is usually characterized by a decrease in training volume, which may lead to detraining and declines in physical fitness. The aim of this study was to examine the effects of a structured transitional training program on anthropometric characteristics, aerobic capacity, and jumping performance in young soccer players. Twenty-three under-17 players participated in the study and, following a two-week period of training cessation, completed a three-week program that included aerobic training three times per week (continuous and interval running sessions) and strength progressive resistance training twice per week. Pre- and post-intervention measurements were analyzed using paired-samples *t*-tests, with statistical significance set at *p* < 0.05. The results revealed significant reductions in body fat percentage (*p* = 0.016, d = 0.547), body fat mass (*p* = 0.018, d = 0.535), and resting systolic blood pressure (*p* = 0.024, d = 0.507). Additionally, time to reach the anaerobic threshold (*p* = 0.022, d = −0.515) and movement speed at the anaerobic threshold (*p* = 0.029, d = −0.487) significantly increased. No significant changes were observed in the remaining variables. These findings indicate that a three-week transition-period training program combining structured aerobic running drills with progressive resistance training can induce favorable adaptations in selected anthropometric and physiological parameters in youth soccer players. However, the lack of a control group should be considered when interpreting the magnitude of the program’s effects.

## 1. Introduction

Football is widely practiced by millions of athletes of both sexes [1,2]. It is a high-intensity intermittent sport lasting 90 min, where performance is multifactorial [3]. More specifically, a player’s performance depends on their physical and mental abilities, technical skills, and tactical knowledge [3]. All of these factors are developed through a long-term plan, so that the player, in early adulthood, can maximize their performance.

Technological development over the past decades has significantly contributed to improvements in physical conditioning [4]. Technologies have been developed that allow the monitoring of training and match load (e.g., global positioning system, GPS), which has led to the creation of player match profiles and the improvement of the training process [5]. According to the technical reports issued by UEFA after the conclusion of its competitions, it appears that the teams participating in the 2024–2025 Champions League ran on average approximately 10 km more than the teams that participated in the 2020–2021 Champions League [6]. Furthermore, it has been reported in the English Premier League that the distance players cover at high speed increased by ~30% between 2006–2012 [7]. The same study noted that the number of sprints also increased by ~85% [7]. Another factor requiring players to maintain excellent physical condition is the large number of matches played during the season. For example, elite-level teams in Europe often compete in more than 50 matches per year [8].

A football team’s yearly macrocycle usually comprises the preparation, competition, and transition phases [9]. The duration of each period depends on the team’s level and the competitions in which it participates. In elite football teams, the preparatory period is generally 4–6 weeks long, followed by a competitive period of roughly 40–44 weeks and a transition period lasting about 4–6 weeks [9].

The transition period is the time between the cessation of team training after the end of the season and the start of pre-season training. This period can be divided into a vacation phase, when players refrain from training, and a phase of individualized training (with personalized programs) aimed at activation and preparation for pre-season. During the transition period, training volume and intensity are typically reduced. As a result, physical fitness components such as endurance, strength, and speed are negatively affected, a phenomenon known as detraining [10,11]. To restore an athlete’s aerobic capacity to the levels prior to the onset of deconditioning, a period longer than 4 weeks may be required [12]. As can be understood, this time could be saved and used by the coaching staff for the development of the team’s technical and tactical actions. Therefore, the goal of the coaching staff is to maintain players’ physical condition or at least minimize its decline during this period.

The existing body of literature suggests that research investigating the impact of transitional programs on soccer players is limited and mainly concerns professional adult players [13,14,15,16]. Some of these studies observed that transitional programs failed to limit the negative effects of detraining [13,14], whereas in other studies the implementation of such programs reduced the detraining in physical fitness capacities and anthropometric characteristics [15].

In recent decades, special attention has been paid to the training process during developmental ages. Clubs aim to produce well-rounded players capable of competing at the highest level from a young age. For youth players, the transition period provides an excellent opportunity to improve performance through individualized programs, free from the pressure of competition. However, in youth as well, reduced training volume and intensity during the transition period may negatively affect physical capacities [17]. The existing literature examining the effects of specific training programs during the transition period remains limited [17,18]. Ιn a recent study [17] on soccer players under the age of 18, a four-week intervention program was implemented, consisting of three training sessions aimed at improving aerobic capacity and two sessions focused on strength development. The researchers observed that the program limited the negative effects that typically occur due to detraining during the transition period. From the review of the literature, the above study appears to be the only one that examines the effect of an intervention program during the transition period in young soccer players.

The present study aimed to examine the effects of a targeted transition program on the aerobic capacity and jumping performance of young football players. We hypothesize that the program will maintain both aerobic capacity and jumping ability levels in athletes. In the present study, aerobic capacity and jumping ability were selected as the primary variables of interest due to their direct relevance to the specific demands of the transition period in elite soccer. Aerobic capacity is a key focus for soccer teams during the preparation period, as it underpins the ability to sustain high-intensity efforts throughout training sessions and competitive matches. Jumping ability, on the other hand, serves as a practical indicator of an athlete’s explosive power, which is critical for performance in actions such as sprinting, dueling, and aerial challenges. By prioritizing these two components, the study aims to capture both endurance and power adaptations that are most likely to be affected during periods of reduced or modified training, providing meaningful insights into the effectiveness of transitional training strategies.

## 2. Materials and Methods

### 2.1. Study Design

**Figure 1:**
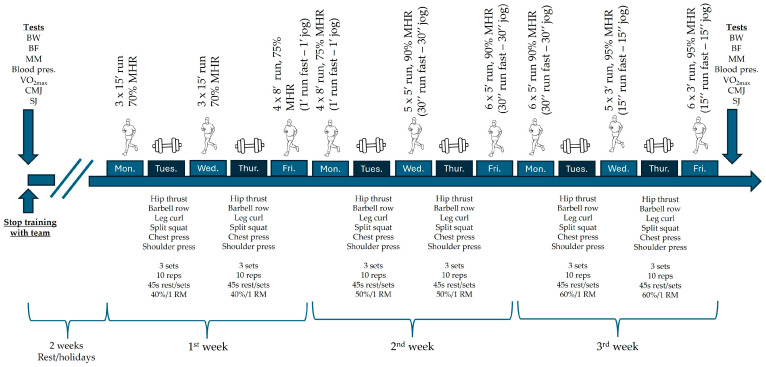
Study design. Note: BW, body weight; BF, body fat; MM, muscle mass; Blood pres., blood pressure; CMJ, countermovement jump; SJ, squat jump, MHR, heart rate max; Reps, repetitions; RM, repetition maximum.

The competitive season ended in mid-May, while team training sessions stopped at the end of June. One week before stopping team training and without any modification to the training load, the players attended the laboratory to undergo anthropometric measurements, and assessment of aerobic capacity and jumping ability. Following this, the players had a two-week vacation period without any scheduled athletic activities. Finally, they undertook the three-week transitional program under the guidance of the coaching staff. Abstinence from exercise during the two-week break, as well as adherence to the program, was ensured through self-reported statements by the players. After completing this period, the players were reassessed in the laboratory using the same tests as in the first evaluation, in order to begin the preparation phase. Study design is presented in Figure 1. The absence of a control group limits the ability to evaluate the magnitude of the transitional program’s effect.

### 2.2. Participants

The study involved 23 soccer players under the age of 17 (15.76 ± 0.58 years, training age 10.30 ± 1.49 years (95% CI: 9.70–10.91), height 1.76 ± 0.08 m (95% CI: 1.73–1.79)), all members of the same soccer team. The team participated in the national championship and, during the year the study was conducted, finished in first place in the standings. Following the classification outlined in the literature [19], these athletes are considered category 3–4 (highly trained/national level–elite/international level), as six participants are members of their national age-group team, and the team competes in at least two international tournaments annually.

Eligibility criteria for participation included: not having an injury during the past month, not taking medication, completing at least 90% of the planned training sessions, and not engaging in additional training beyond the team’s program. The players and their guardians were informed about the study procedures and signed an informed consent form. The study was conducted in accordance with the Declaration of Helsinki and was approved by the institutional review board. Player characteristics are presented in Table 1.

### 2.3. Anthropometric Measurements

Measurements of body weight, body fat, and muscle mass were performed with a TANITA DC-360 digital scale (Tanita Corporation, Tokyo, Japan). The scale estimates body composition through bioelectrical impedance analysis. Anthropometric measurements were conducted in the morning, and the players were instructed to ensure adequate hydration and to avoid food and caffeine intake prior to the assessment. A Seca 220e stadiometer (Seca, Hamburg, Germany) with 0.1 cm precision was used to measure body height. During anthropometric measurements, the players were barefoot and wore only their underwear.

### 2.4. Blood Pressure

For the measurement of arterial blood pressure, upon arrival at the laboratory, the participants were taken to a secluded room where they remained lying in the supine position for 5 min. Subsequently, the sphygmomanometer (M7, Omron, Vernon Hills, IL, USA) was placed in the middle of the cuff at the level of the right atrium of their heart for the recording of blood pressure. Two measurements were performed, and the average was used in the study.

### 2.5. Assessment of Maturity Status

For the assessment of athletes’ biological maturation, the calculation of age at peak height velocity (PHV) was used [20]. For this purpose, the following equation proposed by Moore et al. (Maturity offset = −7.999994 + (0.0036124 × (age × height))) [20] was applied. In this equation, the athlete’s current chronological age and height are used, and it yields the number of years before or after the age of occurrence of PHV. Subsequently, the football players were categorized as early maturers if they reached PHV at <13 years of age, as normal maturers if PHV occurred at 13–15 years, and as late maturers if PHV occurred at >15 years [21].

### 2.6. Laboratory VO_2_max Measurement

To evaluate aerobic capacity, a maximal treadmill test was conducted (Pulsar; h/p/Cosmos, Nussdorf-Traunstein, Germany). The protocol started at 8 km/h with a 0% gradient. Speed increments of 2 km/h were applied every two minutes until the treadmill reached 12 km/h. From 14 km/h onwards, the treadmill incline increased to 2%, while speed continued to increase every minute until exhaustion.

The breath-by-breath metabolic system (Quark CPET, COSMED, Rome, Italy) was calibrated prior to testing using a 2.0 L calibration syringe and reference gases. VO_2_max was defined as the peak value recorded across a minimum of five consecutive breaths during maximal exercise. Heart rate was continuously measured with a Polar Team Pro monitor (10 Hz, Kempele, Finland).

The measurement of VO_2_max was considered valid when a minimum of three criteria were met, including: (a) final-minute heart rate exceeding 95% of the predicted maximum (220 − age), and (b) a plateau in VO_2_ despite increasing treadmill speed, defined as a change of less than 150 mL/min, (c) respiratory exchange ratio (RER = VCO_2_/VO_2_) ≥ 1.1, (d) voluntary termination of the test despite verbal encouragement, and (e) a high perceived exertion (RPE > 17) on the Borg scale [22,23].

### 2.7. Countermovement Jump (CMJ) and Squat Jump (SJ)

Jumping ability was assessed using the OptoJump optical measurement system (Microgate, Bolzano, Italy) [24], with the players performing both the countermovement jump (CMJ) and the squat jump (SJ). For the CMJ, participants began standing with their hands on their hips, performed a quick descent into a squat, and then jumped as high as possible. In the SJ, starting from a static 90° squat position, players executed a maximal vertical jump without prior downward movement. In cases of incorrect technique, a 30 s rest was given before repeating the attempt. Each player performed two trials per jump with 30 s of rest between attempts, and the highest value was used for analysis. The players were familiar with the jumping techniques used in the assessment tests, as these were part of their physical fitness evaluation conducted three times per year.

### 2.8. Transitional Phase Training Program

The transitional phase program included both aerobic and strength exercises. Specifically, the aerobic program was performed three times per week (Monday, Wednesday, Friday), while the strength program was performed twice per week (Tuesday, Thursday).

Aerobic training lasted 35–50 min, preceded by a 10 min warm-up including running drills and mobility exercises. The initial sessions used continuous training, while later sessions employed high-intensity interval training. In total, nine aerobic sessions were completed.

Strength training included six exercises (hip thrust, rowing, bench press, leg curl, split squat, shoulder press). Training intensity was set at 40% of 1 RM in week 1, 50% in week 2, and 60% in week 3. The program consisted of 3 sets of 10 repetitions each, with 45 s rest between sets. A total of six strength training sessions were conducted, each lasting approximately 60 min. Before strength training, a 15 min warm-up was performed, including cycling, stretching, and light repetitions of the programmed exercises at 20% of 1 RM.

The one-repetition maximum (1 RM) testing for the selected exercises was conducted according to standardized procedures, with the load gradually increased until a single repetition could be performed with proper form. Participants rested for 3–5 min between attempts [25,26]. For exercises where direct determination of the 1 RM was not feasible, submaximal sets (8–10 repetitions) were performed, and the 1 RM was then calculated using validated prediction equations [27]. The determination of one-repetition maximum in the strength exercises was initially performed at the beginning of the preparation period, and the values were subsequently updated every four months. In cases where participants were unable to complete any of the scheduled training sessions, they were required to report it. However, all participants completed all the training sessions as planned. The participants were informed that they should refrain from any other sporting activities or training sessions during the study period. The program and its contents are presented in Figure 1.

### 2.9. Statistical Analysis

A priori sample size estimation was performed using G*Power (version 3.1) [28,29] for a two-tailed paired-samples *t*-test. The sample size calculation was based on an assumed medium-to-large effect size (ES = 0.65), a significance level of α = 0.05, and a desired statistical power of 0.80. The results indicated that at least 21 participants would be required to detect a significant within-subject effect. The present investigation included 23 soccer players, thus exceeding the required sample size and ensuring sufficient statistical power. Data were analyzed using JASP (version 0.19.3), Jamovi (version 2.6), and SPSS (version 29) [30,31,32]. Descriptive statistics were expressed as mean ± standard deviation, standard error of the mean (SEM), and 95% confidence intervals. The normality of data distributions was evaluated through the Shapiro–Wilk test and visual inspection of histograms and Q–Q plots. To test pre- to post-intervention differences, paired-samples *t*-tests were applied. Cohen’s d [33] was used to calculate effect sizes, with values of 0.20, 0.50, and 0.80 representing small, medium, and large effects, respectively. Significance was defined as *p* < 0.05.

## 3. Results

**Figure 2:**
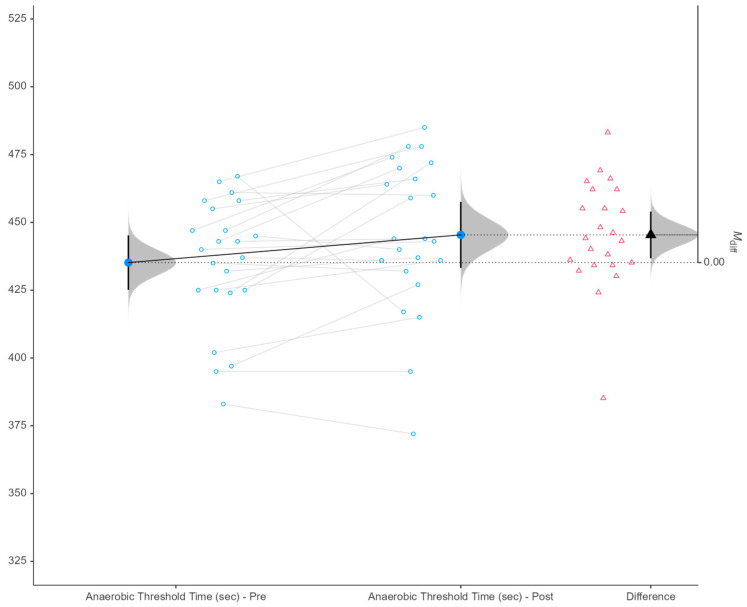
Paired raincloud plot of anaerobic threshold time (sec) pre- and post-intervention (*p* = 0.022). Note: Pre = pre-intervention; Post = post-intervention; Δ = difference (Post–Pre); Mdiff = mean difference; CI = confidence interval. Blue dots represent individual values connected by grey lines across conditions. Red triangles indicate individual Δ scores. Half-violin plots illustrate the distribution of values, while black markers denote group means with 95% confidence intervals (95% CI).

**Figure 3:**
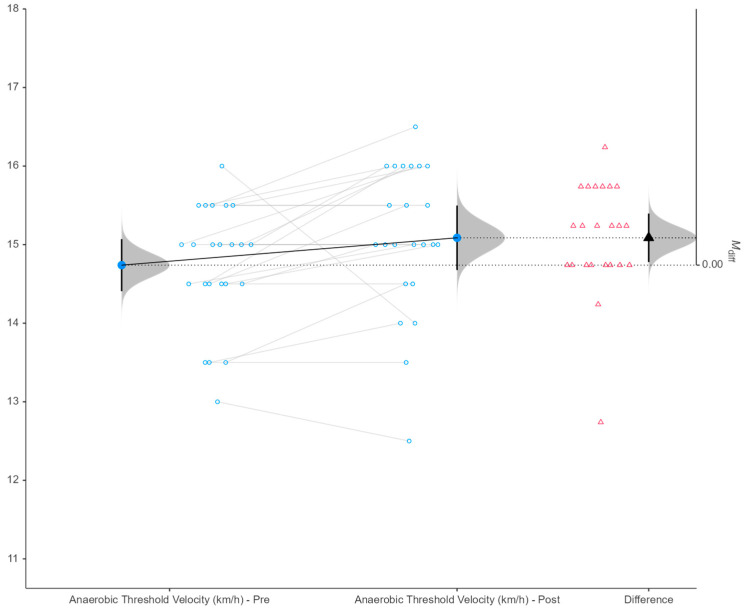
Paired raincloud plot of anaerobic threshold velocity (km/h) pre- and post-intervention (*p* = 0.029). Note: Pre = pre-intervention; Post = post-intervention; Δ = difference (Post–Pre); Mdiff = mean difference; CI = confidence interval. Blue dots represent individual values connected by grey lines across conditions. Red triangles indicate individual Δ scores. Half-violin plots illustrate the distribution of values, while black markers denote group means with 95% confidence intervals (95% CI).

Descriptive statistics for pre- and post-intervention measurements are summarized in Table 1. Paired-samples *t*-tests were used to evaluate changes within participants over the intervention period (Table 2), and revealed significant pre-to-post differences in several anthropometric and physiological variables. Body mass index (BMI) significantly decreased following the intervention (*p* = 0.049, d = 0.434, small effect), alongside reductions in body fat percentage (*p* = 0.016, d = 0.547, moderate effect) and fat mass (*p* = 0.018, d = 0.535, moderate effect). Systolic blood pressure was also significantly lower post-intervention (*p* = 0.024, d = 0.507, moderate effect). In terms of performance-related outcomes, anaerobic threshold time improved significantly (*p* = 0.022, d = −0.515, moderate effect; Figure 2), as did anaerobic threshold velocity (*p* = 0.029, d = −0.487, small-to-moderate effect; Figure 3). No other anthropometric, physiological, or performance variables demonstrated significant pre-to-post differences (Table 2). For these variables, effect sizes were generally trivial to small, with wide 95% confidence intervals (approximately −0.645 to 0.75), particularly for maximal aerobic capacity indices (VO_2_max, maximal exercise time), cardiovascular measures (heart rate variables), and jump performance (CMJ, SJ), indicating substantial inter-individual variability (Table 2). Subsequently, the intervention produced small-to-moderate improvements in body composition, blood pressure, and anaerobic threshold performance, while no significant changes were observed in maximal exercise capacity or jump performance. From the results concerning the biological maturation of the football players, it was observed that 10% of the participants were in normal maturation and the remaining 90% in late maturation.

## 4. Discussion

The results of the present study provide some support for the initial hypotheses. The transitional program followed by the young soccer players was associated with reductions in body fat percentage, fat mass, and systolic blood pressure, as well as modest increases in exercise time until the anaerobic threshold and movement speed at that threshold. Other measured variables were largely maintained, without clear evidence of detraining, although natural variability and the absence of a control group limit definitive conclusions. However, several of these changes were small, approached borderline statistical significance, and may have been influenced by normal variability. In addition, the absence of a control group limits the strength of causal conclusions, suggesting that these findings should be interpreted cautiously.

It has been previously reported that the transitional period is typically characterized by a substantial reduction or even temporary cessation of training load, which may lead to negative effects on players’ physical condition [34,35]. These detraining-related effects have been shown to affect multiple physiological systems, including anthropometric characteristics, neuromuscular function, and the cardiovascular system [34,35]. From a cardiovascular perspective, the absence of adequate training stimuli is associated with reductions in stroke volume and maximal cardiac output, as well as increases in arterial blood pressure [34,35]. Contrary to these commonly reported trends, the present study observed a reduction in systolic blood pressure following the implementation of the intervention program. This finding may indicate that the inclusion of structured training stimuli during the transitional period was sufficient to maintain, or even improve, certain aspects of cardiovascular regulation despite the overall reduction in training load. Similar outcomes have been reported in a recent study by Isbilir et al. [15], who also observed favorable changes in blood pressure following a transitional intervention. However, to date, there remains a paucity of studies examining cardiovascular variables, and blood pressure in particular, in response to transitional programs in soccer players. Therefore, these findings should be interpreted with caution, as they may be influenced by factors such as individual variability, baseline cardiovascular status, or measurement conditions. Nonetheless, the observed reduction in systolic blood pressure suggests that appropriately designed transitional programs may play a protective role in maintaining cardiovascular health during this phase, highlighting the need for further research to confirm and expand upon these preliminary observations.

Examining the effect of the transitional program on anthropometric characteristics, we observed a reduction in body fat percentage and fat mass, while no changes were detected in body mass or muscle mass. This finding is particularly relevant from a practical perspective, as increases in body fat and concurrent losses in muscle mass during the transitional period would require the coaching staff to allocate considerable time during the subsequent preparation phase not only to reduce excess fat but, more importantly, to restore lean tissue. A review of the literature examining the effects of transitional programs in soccer players indicates that the majority of studies have focused on adult professional populations [13,14,15,36]. In one of the earliest investigations [37], conducted in professional soccer players over a four-week transitional program, both the control and experimental groups exhibited increases in body mass and body fat percentage, although these changes were less pronounced in the experimental group. Similarly, in two subsequent studies [14,36], players refrained from structured training for two weeks and then completed four weeks of low-intensity aerobic running three times per week, resulting in increases in both body mass and body fat percentage. Comparable increases were also reported in a more recent study [38]. In contrast, Joo et al. [13] demonstrated that in semi-professional soccer players, anthropometric characteristics returned to baseline values following a five-week transitional period consisting of two weeks of rest and three weeks of high-intensity training. Supporting these findings, a three-week program incorporating flywheel resistance training after two weeks of rest was shown to restore body mass and body fat percentage [16]. More recently, Isbilir et al. [15] reported maintenance of anthropometric characteristics in professional soccer players, while similar outcomes were observed in youth players following a four-week transitional program implemented after two weeks of rest [17]. Collectively, the discrepancies observed across studies are likely attributable to differences in program design, particularly with respect to training intensity and the inclusion of strength-based stimuli. Transitional programs characterized by reduced training volume but relatively higher intensity appear to be more effective in preserving, or even improving, anthropometric characteristics, highlighting the importance of carefully structured interventions during this phase.

In the present study, VO_2_max levels were maintained after the 3-week exercise program. However, it was observed that the speed at the anaerobic threshold of the soccer players improved, and the exercise time until the anaerobic threshold also increased. In the literature, most studies report a decrease in VO_2_max or other indicators of aerobic capacity. Specifically, in two studies where low-intensity aerobic exercise was performed for 4 weeks following 2 weeks of detraining, VO_2_max could not be maintained [14,36]. Similar findings have been reported in studies applying combined aerobic [37], anaerobic [38], and strength training. In contrast, two recent studies [15,17] conducted in professional and youth soccer players reported improvements in VO_2_max. In the study by Isbilir et al. [15], which was conducted on young professionals, a decrease in the time to the anaerobic threshold as well as a reduction in the movement speed of the soccer players at the anaerobic threshold was observed, which the researchers attributed to the specialization of the training stimulus. In those studies, after 2 weeks without systematic training, transitional programs including both aerobic (continuous and interval) and strength exercises were implemented. A lack of changes was also reported in another study that compared three different transitional programs [39]. Previous research has suggested that high-intensity interval training every second week may help maintain aerobic capacity in soccer players [12]. The intensity of running drills, combined with overall training volume, seems to account for differences in results across studies. From the above, it can be observed that the findings of the studies are not consistent. These discrepancies can be attributed to various factors related to study design. More specifically, the type and characteristics of the exercises included in the transitional program (i.e., training volume and intensity) may influence its outcomes. Positive effects have been observed in programs that included higher-intensity exercises [17]. In addition, the competitive level of the soccer players may affect the results. It is well known that the more developed a physical capacity is, the more difficult it becomes to further improve it; at the same time, insufficient training stimuli may lead to a greater decline. Notably, studies reporting reductions in VO_2_max were conducted in professional soccer players [39]. Furthermore, other factors such as the maturation status of youth players, training age, and the assessment protocols used may also contribute to differences in study outcomes. Therefore, these results should be interpreted cautiously, acknowledging that preserved VO_2_max does not necessarily imply maximal adaptation, but rather maintenance within the context of transitional training programs. In addition to the group-level improvements observed in anaerobic threshold time and velocity, the individual response plots (Figure 2 and Figure 3) reveal a small number of players exhibiting pronounced negative changes across the intervention period. These extreme values indicate substantial inter-individual variability in training responsiveness, which is well documented in elite youth soccer populations. Such declines do not appear to reflect a systematic effect of the intervention, but rather isolated responses that may be influenced by factors inherent to applied high-performance settings, including accumulated fatigue from preceding competitive demands, individual differences in recovery capacity, minor illness or injury, or variability in adherence to the prescribed training stimulus. Moreover, given that 90% of the participants were classified as late maturers, non-linear or delayed adaptations to aerobic and speed-related stimuli may partially explain the heterogeneous responses observed. Importantly, these individual declines were retained in the analysis to preserve ecological validity and avoid selective data exclusion. Their presence is reflected in the moderate effect sizes and relatively wide confidence intervals reported, underscoring the importance of interpreting transitional training outcomes not only at the group level but also in the context of individual athlete responses.

With regard to jumping ability, the present study observed its maintenance after the transitional program. In the literature, most studies report a decrease in jumping performance during similar periods. Koundourakis et al. [14,36] reported reductions in both squat jump (SJ) and countermovement jump (CMJ) performance, which were attributed to the exclusive use of low-intensity aerobic training during the transitional phase. In contrast, the findings of the present study are in line with those reported by Requena et al. [38], who also observed maintenance of jumping ability following the transitional period. These discrepancies between studies may largely be explained by differences in program design and training content. Specifically, Koundourakis et al. [14,36] implemented programs focused solely on aerobic activities, whereas the present study, as well as those by Requena et al. [38] and Isbilir et al. [15], incorporated strength-based exercises. The inclusion of strength training may help preserve neuromuscular function by maintaining the efficiency of the stretch–shortening cycle, which plays a crucial role in jumping performance. Moreover, strength-oriented stimuli during the transitional period may counteract detraining effects commonly associated with reduced training loads. Supporting this interpretation, recent studies in youth soccer players have shown either no changes in SJ performance accompanied by a decline in CMJ [17], or complete maintenance of CMJ performance [39]. Collectively, these findings suggest that the inclusion of strength training during the transitional period may be a key factor in mitigating declines in jumping ability and maintaining explosive performance capacities in soccer players. As mentioned above, in the present study, 10% of the participants were classified as being in normal maturation, while the remaining 90% were in late maturation, resulting in a highly skewed maturity distribution. This imbalance may have important implications for training responsiveness, as players at different stages of biological development can exhibit distinct adaptations to the same training stimulus. In particular, young athletes with advanced biological maturation gain greater benefits from neuromuscular training and strength training. Specifically, late-maturing athletes may respond differently in terms of strength, power, and aerobic capacity compared to their normally matured peers. Furthermore, the skewed distribution limits the generalizability of the findings, as the results primarily reflect the characteristics and adaptations of late-maturing players, and may not be directly applicable to a more balanced or younger population. Addressing this aspect is essential for interpreting the study outcomes and for applying the findings to broader groups of youth soccer players.

This study presents some limitations that should be considered when interpreting the findings. The absence of a control group represents a notable limitation of the present study. Without a control condition, it is not possible to conclusively attribute the observed changes solely to the implemented intervention, as other uncontrolled factors—such as natural performance fluctuations, motivational influences, or external training stimuli—may have contributed to the results. This limitation consequently weakens the ability to establish a definitive causal relationship between the intervention and the measured outcomes. Nevertheless, the inclusion of a non-training control group was not considered feasible within this population. The participants were highly trained youth athletes (category 3–4), several of whom were members of their national age-group teams and regularly engaged in international competition. In contemporary elite youth sport settings, even during the transition or off-season period, complete training cessation is uncommon and generally discouraged due to the risk of detraining and negative developmental consequences. As such, assigning elite athletes to a passive control condition would not reflect current high-performance practice and is more typical of non-elite or recreational populations. From a statistical perspective, the absence of a control group combined with the inclusion of multiple dependent variables may limit the analytical depth of the current approach. Although the applied statistical procedures were appropriate for the study design and research questions, the use of alternative analytical strategies or procedures for controlling type I error could provide a more comprehensive evaluation of the data structure and inter-variable relationships (Supplementary Table S1). In addition, training adherence during the intervention period was not objectively monitored. Although participants were provided with standardized training guidelines, variability in compliance and execution cannot be excluded. Differences in individual adherence, as well as uncontrolled variations in training intensity, volume, or recovery strategies, may have meaningfully influenced the observed outcomes. Furthermore, individual responsiveness to training may have varied substantially among players due to differences in biological maturation, training history, or baseline fitness levels, which were not specifically controlled for in the present design. The lack of objective monitoring tools (e.g., heart rate, GPS data, or session rating of perceived exertion) limits the ability to verify the actual training load imposed and to precisely relate training stimuli to observed adaptations.

The absence of mid-point measurements, specifically after the detraining period but prior to the intervention, represents another limitation of the study. Without these measurements, it is not possible to quantify the exact extent of performance decline during the detraining period, which limits the ability to precisely determine the magnitude of the intervention’s restorative effects. Consequently, the interpretation of the intervention’s impact relies on comparisons between pre- and post-intervention assessments, potentially underestimating or overestimating the true effects. Future studies should include mid-point assessments to allow for a more accurate evaluation of both detraining-related declines and subsequent intervention-induced improvements. The study sample was limited to a single soccer team, which may restrict the generalizability of the findings. Characteristics specific to this team, such as training culture, coaching style, and player demographics, could have influenced the observed responses to the intervention. Consequently, the results may not fully represent the outcomes that might be observed in teams with different competitive levels, age groups, or training environments. Future research involving multiple teams or more diverse populations is necessary to determine whether the intervention’s effects are consistent across different contexts and to enhance the external validity of the findings.

Another limitation was the limited range of physical fitness variables assessed. Specifically, the inclusion of additional measures such as maximal strength and agility would have contributed to a more comprehensive understanding of changes in the soccer players’ physical condition and the overall effectiveness of the intervention.

## 5. Conclusions

The findings of the present investigation suggest that a three-week transitional training program, combining structured aerobic-based running drills with progressive resistance training, was associated with modest changes in selected anthropometric and physiological markers in youth soccer players. Observed changes included small reductions in body mass index, body fat percentage, fat mass, and systolic blood pressure, as well as improvements in anaerobic threshold–related variables, such as threshold time and running velocity. However, several of these changes were small or borderline significant and may have been influenced by natural variability. Furthermore, the highly skewed maturity distribution of the sample, with the majority of players classified as late maturers, limits the generalizability of the findings. Therefore, while the results suggest that the implemented program may help maintain or modestly improve certain health- and performance-related parameters during the transitional period, the absence of a control group and other methodological limitations require that these conclusions be interpreted with caution.

## Figures and Tables

**Table 1:** Descriptive Statistics of Anthropometric, Physiological, and Performance Variables Pre- and Post-Intervention.

	Pre				Post			
Variable	Mean ± SD	SEM	95% CI Lower	95% CI Upper	Mean ± SD	SEM	95% CI Lower	95% CI Upper
BMI *	21.64 ± 0.87	0.18	21.29	22.00	21.45 ± 0.80	0.16	21.12	21.78
Body Fat Percentage (%) *	10.77 ± 1.91	0.40	9.99	11.55	9.99 ± 2.07	0.43	9.14	10.84
Muscle mass (kg)	56.87 ± 5.68	1.18	54.55	59.19	57.10 ± 5.79	1.20	54.73	59.47
Fat mass (kg) *	7.21 ± 1.53	0.32	6.58	7.84	6.69 ± 1.68	0.35	6.00	7.37
Body weight (kg)	66.95 6.54	1.37	64.27	69.62	66.81 6.88	1.44	63.99	69.62
Resting Heart Rate (bpm)	70.26 ± 9.54	1.99	66.36	74.16	72.39 ± 10.47	2.18	68.11	76.67
Systolic (mmHg) *	126.9 ± 8.6	1.8	123.3	130.4	120.8 ± 10.5	2.2	116.5	125.1
Diastolic (mmHg)	68.6 ± 8.6	1.8	65.1	72.1	65.7 ± 7.0	1.4	62.9	68.6
Anaerobic Threshold Time (sec) *	435.17 ± 23.22	4.84	425.68	444.66	445.39 ± 28.16	5.87	433.88	456.90
Anaerobic Threshold Heart Rate (bpm)	181.87 ± 8.37	1.74	178.44	185.29	182.26 ± 4.66	0.97	180.35	184.16
Anaerobic Threshold Velocity (km/h) *	14.73 ± 0.76	0.16	14.42	15.05	15.08 ± 0.94	0.19	14.69	15.47
Anaerobic Threshold VO_2_ (mL/kg/min)	52.73 ± 3.75	0.78	51.19	54.26	51.60 ± 5.45	1.13	49.37	53.83
Maximal Exercise Time (sec)	521.69 ± 38.27	7.98	506.05	537.33	526.43 ± 29.53	6.15	514.36	538.50
Maximal Heart Rate (bpm)	198.17 ± 8.75	1.82	194.59	201.75	199.34 ± 8.36	1.74	195.92	202.76
Maximal Velocity (km/h)	17.67 ± 1.30	0.27	17.14	18.20	17.76 ± 0.98	0.20	17.35	18.16
Max VO_2_ (mL/kg/min)	58.35 ± 3.48	0.72	56.93	59.78	58.12 ± 4.03	0.84	56.47	59.77
Max VO_2_ (mL/min)	3904.47 ± 426.37	88.9	3730.22	4078.73	3879.60 ± 445.29	92.85	3697.62	4061.59
Respiratory Exchange Ratio (RER)	1.16 ± 0.04	0.01	1.14	1.17	1.17 ± 0.03	0.01	1.15	1.18
CMJ (cm)	37.53 ± 4.04	0.84	35.87	39.18	37.35 ± 4.51	0.94	35.50	39.19
SJ (cm)	36.25 ± 4.32	0.90	34.48	38.02	35.53 ± 3.93	0.82	33.92	37.14

Note: Pre = pre-intervention assessment; Post = post-intervention assessment; Mean ± SD = arithmetic mean and standard deviation; SEM = standard error of the mean; CI = confidence interval; BMI = body mass index; bpm = beats per minute; mmHg = millimetres of mercury; VO_2_ = oxygen uptake; RER = respiratory exchange ratio; CMJ = countermovement jump; SJ = squat jump. * *p* < 0.05 indicates statistically significant pre–post differences.

**Table 2:** Results of Paired-Samples *t*-Tests for Within-Subject Comparisons Between Pre- and Post-Intervention Assessments.

Pre- vs. Post-Intervention Comparison	*t*	*p*	Cohen’s d	SE Cohen’s d	95% CI for Cohen’s d	
					Lower	Upper
BMI	2.081	0.049 *	0.434	0.115	0.001	0.858
Body Fat Percentage (%)	2.621	0.016 *	0.547	0.159	0.102	0.980
Muscle mass (kg)	−0.869	0.394	−0.181	0.047	−0.591	0.233
Fat mass (kg)	2.564	0.018 *	0.535	0.135	0.092	0.967
Body weight (kg)	0.487	0.631	0.102	0.041	−0.309	0.510
Resting Heart Rate (bpm)	−1.007	0.325	−0.210	0.213	−0.621	0.206
Systolic (mmHg)	2.431	0.024 *	0.507	0.273	0.067	0.937
Diastolic (mmHg)	1.568	0.131	0.327	0.237	−0.096	0.743
Anaerobic Threshold Time (sec)	−2.469	0.022 *	−0.515	0.167	−0.945	−0.074
Anaerobic Threshold Heart Rate (bpm)	−0.258	0.799	−0.054	0.208	−0.462	0.356
Anaerobic Threshold Velocity (km/h)	−2.336	0.029 *	−0.487	0.178	−0.915	−0.049
Anaerobic Threshold VO_2_ (mL/kg/min)	1.105	0.281	0.230	0.214	−0.186	0.642
Maximal Exercise Time (sec)	−1.121	0.275	−0.234	0.115	−0.645	0.183
Maximal Heart Rate (bpm)	−0.982	0.337	−0.205	0.141	−0.616	0.211
Maximal Velocity (km/h)	−0.569	0.575	−0.119	0.122	−0.527	0.293
Max VO_2_ (mL/kg/min)	0.331	0.743	0.069	0.183	−0.341	0.478
Max VO_2_ (mL/min)	0.573	0.573	0.119	0.100	−0.292	0.528
Respiratory Exchange Ratio (RER)	−1.007	0.325	−0.210	0.246	−0.621	0.206
CMJ (cm)	0.338	0.738	0.071	0.121	−0.339	0.479
SJ (cm)	1.600	0.124	0.334	0.110	−0.090	0.750

Note: Pre- vs. Post-Intervention Comparison = comparison between pre-intervention and post-intervention measurements; *t* = paired-samples *t* statistic; *p* = significance level; Cohen’s d = standardized effect size; SE = standard error; CI = confidence interval; BMI = body mass index; bpm = beats per minute; mmHg = millimetres of mercury; VO_2_ = oxygen uptake; RER = respiratory exchange ratio; CMJ = countermovement jump; SJ = squat jump. * *p* < 0.05 indicates statistical significance.

## Data Availability

The original contributions presented in the study are included in the article/Supplementary Material, further inquiries can be directed to the corresponding author.

## Supplementary Materials

The following supporting information can be downloaded at: https://www.mdpi.com/article/10.3390/sports14030088/s1, Supplementary Table S1. Repeated-measures ANOVA (Time: Pre vs. Post) and estimated marginal means for variables showing statistically significant pre–post differences in paired-samples *t*-tests.

## References

[1] FIFA (2021). Professional Football. https://publications.fifa.com/en/annual-report-2021/around-fifa/professional-football-2021/.

[2] FIFA Advancing Football. https://inside.fifa.com/advancing-football.

[3] Williams A.M., Ford P.R., Drust B. (2020). Talent identification and development in soccer since the millennium. J. Sports Sci..

[4] Vardakis L., Michailidis Y., Topalidis P., Zelenitsas C., Mandroukas A., Gissis I., Christoulas K., Mavrommatis G., Metaxas T. (2023). Application of a structured training plan on different-length microcycles in soccer—Internal and external load analysis between training weeks and games. Appl. Sci..

[5] Michailidis Y., Stafylidis A., Vardakis L., Kyranoudis A.E., Mittas V., Bilis V., Mandroukas A., Metaxas I., Metaxas T.I. (2025). Influence of Playing Position on the Match Running Performance of Elite U19 Soccer Players in a 1-4-3-3 System. Appl. Sci..

[6] UEFA Technical Reports. https://www.uefatechnicalreports.com/.

[7] Barnes C., Archer D.T., Hogg B., Bush M., Bradley P.S. (2014). The Evolution of Physical and Technical Performance Parameters in the English Premier League. Int. J. Sports Med..

[8] Barca Innovation Hub The Increase in Football Matches Heightens the Risk of Devaluation and Loss of Product Quality. https://barcainnovationhub.fcbarcelona.com/?s=The+Increase+in+Football+Matches+Heightens+the+Risk+of+Devaluation+and+Loss+of+Product+Quality.

[9] Silva J.R., Brito J., Akenhead R., Nassis G.P. (2016). The Transition Period in Soccer: AWindow of Opportunity. Sports Med..

[10] Vassilis S., Yiannis M., Athanasios M., Dimitrios M., Ioannis G., Thomas M. (2019). Effect of a 4-Week Detraining Period Followed by a 4-Week Strength Program on Isokinetic Strength in Elite Youth Soccer Players. J. Exerc. Rehabil..

[11] Clemente F.M., Ramirez-Campillo R., Sarmento H. (2021). Detrimental Effects of the Off-Season in Soccer Players: A Systematic Review and Meta-Analysis. Sports Med..

[12] Slettaløkken G., Rønnestad B.R. (2014). High-intensity interval training every second week maintains VO_2_max in soccer players during off-season. J. Strength Cond. Res..

[13] Joo C.H. (2018). The effects of short term detraining and retraining on physical fitness in elite soccer players. PLoS ONE.

[14] Koundourakis N.E., Androulakis N.E., Malliaraki N., Margioris A.N. (2014). Vitamin D and exercise performance in professional soccer players. PLoS ONE.

[15] Isbilir M., Stafylidis A., Michailidis Y., Mandroukas A., Antoniou G., Semaltianou E., Mittas V., Ispirlidis I., Metaxas T.I. (2025). Pre- and Post-Test Evaluation of a Periodized Off-Season Training Program in Professional Footballers. Appl. Sci..

[16] Suarez-Arrones L., Lara-Lopez P., Maldonado R., Torreno N., De Hoyo M., Yuzo Nakamura F., Di Salvo V., Mendez-Villanueva A. (2019). The effects of detraining and retraining periods on fat-mass and fat-free mass in elite male soccer players. PeerJ.

[17] Michailidis Y., Stafylidis A., Mandroukas A., Semaltianou E., Karamousalidis G., Antoniou G., Leftheroudis V., Mittas V., Metaxas T.I. (2025). The Effect of a High-Frequency Exercise Program During the Transition Period in Young Football Players. Sports.

[18] Padron-Cabo A., Lorenzo-Martinez M., De Dios-Alvarez V., Rey E., Solleiro-Duran D. (2025). Effects of a Short-Term Detraining Period on the Physical Fitness in Elite Youth Soccer Players: A Comparison between Chronological Age Groups. J. Strength Cond. Res..

[19] McKay A.K.A., Stellingwerff T., Smith E.S., Martin D.T., Mujika I., Goosey-Tolfrey V.L., Sheppard J., Burke L.M. (2022). Defining Training and Performance Caliber: A Participant Classification Framework. Int. J. Sports Physiol. Perform..

[20] Moore S.A., McKay H.A., Macdonald H., Nettlefold L., Baxter-Jones A.D., Cameron N., Brasher P.M. (2015). Enhancing a Somatic Maturity Prediction Model. Med. Sci. Sports Exerc..

[21] Sherar L.B., Mirwald R.L., Baxter-Jones A.D., Thomis M. (2005). Prediction of adult height using maturity-based cumulative height velocity curves. J. Pediatr..

[22] Brink-Elfegoun T., Kaijser L., Gustafsson T., Ekblom B. (2007). Maximal Oxygen Uptake Is Not Limited by Central Nervous System Governor. J. Appl. Physiol..

[23] Astrand P.O., Rodahl K. (1986). Evaluation of Physical Performance on the Basis of Tests. Textbook of Work Physiology.

[24] Freitas T.T., Pereira L.A., Alcaraz P.E., Arruda A.F., Guerriero A., Azevedo P.H., Loturco I. (2019). Influence of Strength and Power Capacity on Change of Direction Speed and Deficit in Elite Team-Sport Athletes. J. Hum. Kinet..

[25] Baechle T.R., Earle R.W. (2008). Essentials of Strength Training and Conditioning.

[26] Kraemer W.J., Ratamess N.A. (2004). Fundamentals of Resistance Training: Progression and Exercise Prescription. Med. Sci. Sports Exerc..

[27] Reynolds J.M., Gordon T.J., Robergs R.A. (2006). Prediction of One Repetition Maximum Strength from Multiple Repetition Maximum Testing and Anthropometry. J. Strength Cond. Res..

[28] Faul F., Erdfelder E., Buchner A., Lang A.G. (2009). Statistical Power Analyses Using G*Power 3.1: Tests for Correlation and Regression Analyses. Behav. Res. Methods.

[29] Faul F., Erdfelder E., Lang A.G., Buchner A. (2007). G*Power 3: A Flexible Statistical Power Analysis Program for the Social, Behavioral, and Biomedical Sciences. Behav. Res. Methods.

[30] The Jamovi Project (2025). Jamovi.

[31] IBM Corporation (2025). IBM SPSS Statistics for Windows.

[32] JASP Team (2025). JASP.

[33] Cohen J. (1988). Statistical Power Analysis for the Behavioral Sciences.

[34] Mujika I., Padilla S. (2000). Detraining: Loss of training-induced physiological and performance adaptations. Part I Sport Med..

[35] Mujika I., Padilla S. (2000). Detraining: Loss of training-induced physiological and performance adaptations. Part II: Long term insufficient training stimulus. Sports Med..

[36] Koundourakis N.E., Androulakis N.E., Malliaraki N., Tsatsanis C., Venihaki M., Margioris A.N. (2014). Discrepancy between exercise performance, body composition, and sex steroid response after a six-week detraining period in professional soccer players. PLoS ONE.

[37] Sotiropoulos A., Travlos A.K., Gissis I., Souglis A.G., Grezios A. (2009). The effect of a 4-week training regimen on body fat and aerobic capacity of professional soccer players during the transition period. J. Strength Cond. Res..

[38] Requena B., García I., Suárez-Arrones L., De Villarreal E.S., Naranjo Orellana J., Santalla A. (2017). Off-season effects on functional performance, body composition, and blood parameters in top-level professional soccer players. J. Strength Cond. Res..

[39] Liu G., Wang X., Xu Q. (2024). Supervised Offseason Training Programs are able to mitigate the Effects of Detraining in Youth Men Soccer Players Physical Fitness: A Randomized Parallel Controlled Study. J. Sports Sci. Med..

