# Supplementary material for: Fitness Adaptations to a Combined Strength and Aerobic Training Program During the Transition Period in Young Soccer Players
Source: Sports (Basel). 2026 Feb 26;14(3):88. doi: 10.3390/sports14030088 (PMC13029815; doi:10.3390/sports14030088)
Supplement: Supplementary file 1 [file sports-14-00088-s001.zip › sports-4111193-supplementary.docx]

**Supplementary Tables and figures**

**Supplementary Table S1.** Repeated-measures ANOVA (Time: Pre vs. Post) and estimated marginal means for variables showing statistically significant pre–post differences in paired-samples t-tests

- Body mass index (BMI),
- Body fat percentage (%),
- Fat mass (kg),
- Systolic blood pressure (mmHg),
- Anaerobic threshold time (sec),
- Anaerobic threshold velocity (km/h).

| **Variable** | **Pre M (SE)** | **95% CI (Pre)** | **Post M (SE)** | **95% CI (Post)** | **SS_Time** | **SS_Error** | **MS_Time** | **MS_Error** | **F(1,22)** | **p** | **ηp²** |
| --- | --- | --- | --- | --- | --- | --- | --- | --- | --- | --- | --- |
| Body mass index (BMI) | 21.648 (0.183) | [21.269, 22.027] | 21.454 (0.169) | [21.104, 21.804] | 0.435 | 2.212 | 0.435 | 0.101 | 4.331 | .049* | .164 |
| Body fat percentage (%) | 10.774 (0.400) | [9.945, 11.603] | 9.991 (0.433) | [9.093, 10.890] | 7.043 | 22.557 | 7.043 | 1.025 | 6.870 | .016* | .238 |
| Fat mass (kg) | 7.217 (0.320) | [6.553, 7.882] | 6.691 (0.350) | [5.965, 7.418] | 3.183 | 10.652 | 3.183 | 0.484 | 6.574 | .018* | .230 |
| Systolic blood pressure (mmHg)* | 12.691 (0.181) | [12.316, 13.066] | 12.087 (0.220) | [11.631, 12.543] | 4.200 | 15.635 | 4.200 | 0.711 | 5.910 | .024* | .212 |
| Anaerobic threshold time (sec) | 435.174 (4.843) | [425.130, 445.218] | 445.391 (5.873) | [433.211, 457.572] | 1200.543 | 4332.957 | 1200.543 | 196.953 | 6.096 | .022* | .217 |
| Anaerobic threshold velocity (km·h⁻¹) | 14.739 (0.160) | [14.408, 15.071] | 15.087 (0.198) | [14.676, 15.497] | 1.391 | 5.609 | 1.391 | 0.255 | 5.457 | .029* | .199 |

*Note: Repeated-measures ANOVA with Time (Pre vs. Post) as the within-subject factor was conducted only for variables that demonstrated statistically significant pre–post differences in the paired-samples t-tests. This analysis was performed as a confirmatory step to verify the robustness of the original findings. Type III sums of squares were used for all analyses. Pre = pre-intervention assessment; Post = post-intervention assessment; M = marginal mean; SE = standard error of the mean; CI = confidence interval; SS_Time = sum of squares for the within-subject factor Time; SS_Error = sum of squares for the error term; MS_Time = mean square for Time; MS_Error = mean square error; F(1,22) = F statistic with 1 and 22 degrees of freedom; p = significance level; ηp² = partial eta squared. BMI = body mass index; mmHg = millimetres of mercury; sec = seconds; km·h⁻¹ = kilometres per hour. p < .05 indicates statistical significance.*
